# Utility of collagen-derived peptides as markers of organ injury in patients with acute heart failure

**DOI:** 10.1136/openhrt-2019-001041

**Published:** 2020-04-05

**Authors:** Kazuya Nagao, Akinori Tamura, Yukihito Sato, Reo Hata, Yuichi Kawase, Kazushige Kadota, Takahiro Horie, Naoya Sowa, Masataka Nishiga, Koh Ono, Tsukasa Inada, Masaru Tanaka

**Affiliations:** 1 Cardiovascular Center, Osaka Red Cross Hospital, Osaka, Japan; 2 Department of Cardiology, Hyogo Prefectural Amagasaki General Medical Center, Amagasaki, Japan; 3 Department of Cardiology, Kurashiki Central Hospital, Kurashiki, Japan; 4 Department of Cardiovascular Medicine, Kyoto University Graduate School of Medicine, Kyoto, Japan

**Keywords:** heart failure, biomarkers, fibrosis, 7S collagen

## Abstract

**Objective:**

This study aims to investigate the time-dependent prognostic utility of two fibrosis markers representing organ fibrogenesis (N-terminal propeptide of procollagen III (PIIINP) and type IV collagen 7S (P4NP 7S)) in patients with acute heart failure (HF).

**Methods:**

390 patients with acute HF were dichotomised based on the median value of fibrosis markers at discharge. The primary outcome measure was a composite of cardiac death and HF hospitalisation.

**Results:**

P4NP 7S significantly declined during hospitalisation, whereas PIIINP did not. The cumulative 90-day and 365-day incidence of the primary outcome measure was 16.6% vs 16.0% (p=0.42) and 33.3% vs 28.4% (p=0.34) in the patients with high versus low PIIINP; 19.9% vs 13.0% (p=0.04) and 32.3% vs 29.0% (p=0.34) in the patients with high and low P4NP 7S, respectively. After adjusting for confounders, high P4NP 7S correlated with significant excess risk relative to low P4NP 7S for both 90-day and 365-day primary outcome measure (adjusted HR, 1.50; 95% CI, 1.02 to 2.21; p=0.04 and adjusted HR, 1.89; 95% CI, 1.11 to 3.26; p=0.02, respectively), which was driven by significant association of high P4NP 7S with higher incidence of HF hospitalisation. Furthermore, P4NP 7S exhibited an additive value to conventional prognostic factors for predicting 90-day outcome (p=0.038 for net reclassification improvement; p=0.0068 for integrated discrimination improvement). High PIIINP did not correlate with significant excess risk for both 90-day and 365-day outcome.

**Conclusions:**

This study suggests a possible role of P4NP 7S in the risk stratification of patients with acute HF.

**Key questions**

**What is already known about this subject?**

- Systemic venous congestion in heart failure (HF) causes end-organ injury, which adversely affects prognosis. It has been recently reported that the serum 7S domain of the collagen type IV N-terminal propeptide (P4NP 7S), an established marker of liver fibrosis correlated with liver function tests, pulmonary capillary wedge pressure and right ventricular and atrial pressure in patients with HF. In addition, in a single centre exploratory analysis, P4NP 7S exhibited a potential prognostic utility.

**What does this study add?**

- In patients hospitalised for acutely decompensated heart failure (HF), P4NP 7S at discharge correlated with significant excess risk for HF hospitalisation. P4NP 7S was superior to conventional liver function tests and another liver fibrogenic marker, N-terminal propeptide of procollagen III (PIIINP) for the prognostic utility.

**How might this impact on clinical practice?**

- The evaluation of P4NP 7S at discharge might facilitate the identification of those patients at high risk for persistent end-organ injury and subsequent heart failure-related events.

## Introduction

Systemic venous congestion is a hallmark of heart failure (HF).^1^ Fluid retention in HF can involve the end-organs such as lungs, kidneys and liver, causing injury to multiple organs.^2 3^ Decongestion therapy during hospitalisation could improve the signs and symptoms of HF. However, a subset of patients might have residual subclinical congestion even at discharge, contributing to persistent end-organ injury, recurrence of congestion and early readmission.^4^

Fibrogenetic cascade is a common pathological response in numerous tissues. Even in HF, congestion-induced organ injury could evoke a fibrogenetic response.^5^ Recently, we illustrated that the serum 7S domain of the collagen type IV N-terminal propeptide (P4NP 7S), a marker for liver fibrosis used in the patients with primary liver diseases was elevated and reflected extra-cardiac organ injury in patients with dilated cardiomyopathy^6^ and markedly correlated with the severity of haemodynamic congestion in patients with acutely decompensated heart failure (ADHF).^7^ These results suggest that organ fibrosis markers could be used for risk stratification of patients with HF. Indeed, in a single centre exploratory analysis, P4NP 7S exhibited a potential prognostic utility.^7^

Hence, we conducted a prospective cohort study to evaluate in-hospital changes of two collagen markers (N-terminal propeptide of procollagen III (PIIINP) and P4NP 7S) and to confirm the utility of these markers for predicting early and late clinical outcome.

## Methods

The present study is a prospective cohort study enrolling consecutive patients admitted for ADHF at three tertiary referral hospitals in Japan (online supplementary data). We excluded patients if they had acute coronary syndrome, known active neoplasia, active hepatitis or liver cirrhosis, severe renal dysfunction (creatinine >3 mg/dL or under haemodialysis) or overt inflammatory, metabolic or bone disease. We enrolled 403 eligible patients between February 2016 and March 2017. After excluding 13 patients who died during hospitalisation, 390 patients were examined in this study. Of note, patients enrolled in this study did not overlap with those enrolled in our previous study.^6 7^ The study was approved by an institutional review board. All study procedures complied with the ethical principles of the Declaration of Helsinki, and we obtained written informed consent from all patients.

10.1136/openhrt-2019-001041.supp1 Supplementary data

### Sample collection and biomarker measurements

Blood samples, collected at admission and immediately before discharge, were centrifuged, and the serum was transferred to a central laboratory for PIIINP and P4NP 7S measurement. Owing to the limited serum volume, PIIINP was measured in 330 patients on admission and 384 patients at discharge. Other clinical biomarkers, including brain natriuretic peptide (BNP), renal function and LFTs (liver function tests), were simultaneously assessed as a routine clinical practice.

### Definitions of the clinical outcome measures

The prespecified primary outcome measure in this study was a composite of 365-day cardiac death and HF hospitalisation. The secondary outcome measures were individual components of the primary outcome measure and all-cause death. In addition, our prior exploratory analysis and the previous study by others suggested that the prognostic effect of LFTs and collagen markers might be time-dependent and that the impact of these markers on short-term and long-term outcomes might be different.^7 8^ Hence, as prespecified analyses, we also investigated the time-dependent prognostic utility of each collagen marker separately within and beyond 90 days. We defined HF hospitalisation as hospitalisation because of deteriorating HF that required intravenous drug therapy. For individual patients, follow-up began on the day of discharge through 365 days of discharge. Data regarding survival and hospitalisations were collected through review of hospital charts or collected through contact with patients, relatives and/or referring physicians.

### Statistical analysis

Continuous variables are presented as means±SD or medians and IQR. We assessed the between-group significant differences in continuous variables using the Student’s t-test, Mann-Whitney U-test or Wilcoxon matched-pairs signed-rank test, as appropriate. Differences in categorical variables were assessed using the χ^2^ tests. We tested the correlation between clinical parameters using the Spearman’s correlation coefficient. We dichotomised patients based on the median value of each collagen marker at discharge and estimated cumulative incidences of clinical events across PIIINP and P4NP 7S using the Kaplan-Meier method. The differences in cumulative incidences of clinical events were assessed by the log-rank test. We estimated the risks related to high PIIINP and P4NP 7S for the primary and secondary endpoints relative to the low PIIINP and P4NP 7S using the Cox proportional hazard model, which are presented as HR and 95% CI. Clinically relevant factors, including age, sex, ejection fraction (EF) <40% and estimated glomerular filtration rate (eGFR), were incorporated as risk-adjusting variables. We investigated the time-dependent prognostic utility of each collagen marker by the landmark analysis at 90 days after discharge. Surviving patients with HF hospitalisation within 90 days were included for the analysis beyond 90 days. We also evaluated the HR related to the abnormal value of individual LFTs (online supplementary methods). By calculating the c-index from the receiver-operating characteristic analysis, we evaluated the predictive capabilities of the models.^9^ Furthermore, the incremental prognostic utility of PIIINP and P4NP 7S on the top of a reference model including conventional risk factors was measured using the continuous net reclassification improvement (NRI) and integrated discrimination improvement (IDI).^10^ We also added abnormal LFTs to the reference model separately and compared the incremental prognostic utility. The clinically relevant factors and established prognosticators of ADHF incorporated into the reference model were as follows: age, sex, EF<40%, eGFR, sodium <140 mmol/L, haemoglobin and BNP. In this study, all statistical analyses were performed with JMP V.10.0.0 (SAS Institute Inc, Cary, North Carolina, USA), GraphPad Prism V.6.05 (GraphPad Software, Inc, La Jolla, California, USA) and statistical software R (V.3.3.1). We considered p<0.05 as statistically significant.

## Results

### Baseline characteristics

**Figure 1:**
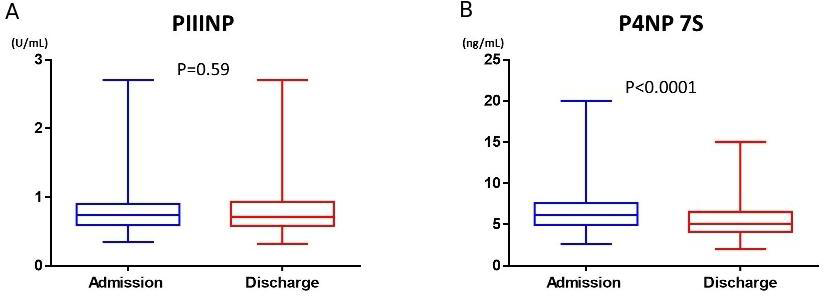
The change of collagen markers during hospitalisation. PIIINP, N-terminal propeptide of procollagen III; P4NP 7S, 7S domain of the collagen type IV N-terminal propeptide.

Table 1 summarises the baseline characteristics of the entire cohort and those dichotomised per the median value of PIIINP and P4NP 7S at discharge. The median in-hospital duration was 14 days. The median PIIINP value at discharge was 0.71 U/mL, which was not significantly different from that on admission (0.74 U/mL, p=0.59; figure 1A). Patients with high PIIINP at discharge were markedly older, had higher EF, tended to be anaemic and less likely to be taking β-blocker and angiotensin-converting enzyme inhibitor or angiotensin receptor blocker than those with low PIIINP (table 1). In addition, BNP and LFTs were not markedly different between those with high and low PIIINP, except for total bilirubin (T-Bil), which was markedly lower in patients with high PIIINP than in those with low PIIINP. The eGFR was lower in patients with high PIIINP than that in patients with low PIIINP. In the entire cohort, weak negative correlation of PIIINP with T-Bil, aspartate aminotransferase (AST), γ-glutamyltransferase (γ-GTP) and modest negative correlation with eGFR were observed (online supplementary table 1).

**Table 1:** Clinical characteristics

Variables	Entire cohort (n=390)	PIIINP at discharge (n=384)			P4NP 7S at discharge (n=390)		
		Low (n=193)	High (n=191)	P value	Low (n=200)	High (n=190)	P value
Clinical characteristics							
Age (years)	76.2±11.5	75.2±11.4	77.5±11.4	0.046	78.8±10.8	73.4±11.7	<0.0001
Male	207 (53)	107 (55)	95 (50)	0.26	94 (47)	113 (59)	0.014
Hypertension	301 (77)	143 (74)	154 (81)	0.13	156 (78)	145 (76)	0.69
Diabetes mellitus	138 (35)	65 (34)	71 (37)	0.5	63 (32)	75 (39)	0.11
Ischaemic heart disease	112 (29)	54 (28)	56 (29)	0.77	64 (32)	48 (29)	0.14
EF<40%	154 (39)	89 (46)	62 (32)	0.0061	74 (37)	80 (42)	0.3
Anaemia	242 (63)	98 (51)	143 (75)	<0.0001	137 (69)	105 (56)	0.008
Medication at discharge							
β-blocker	304 (78)	160 (83)	138 (73)	0.016	152 (76)	152 (80)	0.29
ACEI or ARB	241 (62)	128 (66)	108 (57)	0.049	124 (62)	117 (62)	0.93
MRA	208 (53)	101 (52)	104 (54)	0.68	95 (48)	113 (59)	0.018
Loop diuretics	344 (88)	164 (85)	174 (91)	0.08	172 (86)	172 (91)	0.21
Laboratory tests at discharge							
T-Bil (mg/dL)	0.6 (0.5–0.9)	0.7 (0.5–1)	0.6 (0.4–0.8)	0.0001	0.5 (04–0.7)	0.8 (0.6–1.1)	<0.0001
AST (U/L)	22 (17–29)	23 (18–31)	22 (17–28)	0.055	20 (17–25)	25 (19–32)	<0.0001
ALP (U/L)	234 (190–303)	231 (186–300)	238 (194–317)	0.098	225 (186–288)	243 (198–333)	0.0094
γ-GTP (U/L)	33 (19–67)	37 (22–70)	27 (17–63)	0.66	24 (17–41)	52 (26–90)	<0.0001
Albumin (g/dL)	3.5 (3.1–3.8)	3.5 (3.2–3.9)	3.5 (3–3.75)	0.13	3.5 (3.1–3.8)	3.5 (3.2–3.8)	0.25
eGFR (mL/min/1.73 m^2_)_^	47.2±20.2	53.6±18.6	40.1±19.2	<0.0001	40 (28–58)	48 (34–60)	0.031
BNP (pg/mL)	266 (121–480)	258 (103–443)	268 (131–585)	0.2	207 (96–397)	293 (171–667)	<0.0001
PIIINP (U/mL)	0.71 (0.58–0.93)	0.58 (0.51–0.64)	0.93 (0.81–1.1)	<0.0001	0.67 (0.55–0.86)	0.77 (0.61–1)	0.0019
P4NP 7S (ng/mL)	5.1 (4.1–6.5)	4.7 (3.8–6)	5.6 (4.4–7)	<0.0001	4.1 (3.4–4.6)	6.6 (5.8–7.6)	<0.0001

Values are number (%), mean (SD) or median (IQR).

ACE-I, angiotensin-converting enzyme inhibitor; ALP, alkaline phosphatase; ARB, angiotensin receptor blocker; AST, aspartate aminotransferase; BNP, brain natriuretic peptide; EF, ejection fraction; eGFR, estimated glomerular filtration rate; γ-GTP, γ-glutamyltransferase; MRA, mineral conrticoid receptor antagonist; PIIINP, N-terminal propeptide of procollagen type III; P4NP 7S, 7S domain of the collagen type IV N-terminal propeptide; T-Bil, total bilirubin.

During hospitalisation, P4NP 7S significantly decreased from 6.1 ng/mL on admission to 5.1 ng/mL at discharge (figure 1B). Patients with high P4NP 7S at discharge were markedly younger, more likely to be male, more likely to have lower EF, less likely to be anaemic and more often taking aldosterone antagonists than those with low P4NP 7S. In addition, LFTs and BNP in patients with high P4NP 7S were markedly higher than those in patients with low P4NP 7S, whereas the eGFR was not statistically significant between the two groups. In the entire cohort, P4NP 7S significantly correlated with all LFTs and BNP (online supplementary table 1). PIIINP and P4NP 7S marginally correlated with each other (Spearman’s *r*=0.24; p<0.0001).

### Correlations between changes of markers during hospitalisation

Patients with high PIIINP at discharge were associated with a smaller reduction in AST, γ-GTP, PIIINP and P4NP 7S during hospitalisation, higher reduction in the eGFR and smaller increment in albumin than those with low PIIINP at discharge. In the entire cohort, we observed a weak positive correlation among %DALP, %Dγ-GTP and%ΔPIIINP, whereas a weak negative correlation among %ΔT-Bil, %ΔeGFR and %ΔPIIINP (table 2). Compared with patients with low P4NP 7S at discharge, patients with high P4NP 7S were associated with less recovery of liver injury during hospitalisation as illustrated by a smaller decline in most LFTs and smaller increment in albumin. However, we observed no significant correlation between %ΔP4NP 7S and any %ΔLFTs, except for only a weak correlation with %ΔALP in the entire cohort (table 2). In addition, we observed a smaller decline in the eGFR, BNP, PIIINP and P4NP 7S in patients with high P4NP 7S than those with low P4NP 7S (table 2). Furthermore, %ΔP4NP 7S did not significantly correlate with %ΔeGFR but modestly correlated with %ΔBNP (Spearman’s *r*=0.31; p<0.0001). Of note, %ΔPIIINP and %ΔP4NP 7S only weakly correlated with each other (Spearman’s *r*=0.16; p=0.0047; table 2).

**Table 2:** The correlation between %changes of (%Δ) markers during hospitalisation

	Entire cohort	PIIINP at discharge			P4NP 7S at discharge			%ΔPIIINP		%ΔP4NP 7S	
	Value	Low	High	P value	Low	High	P value	Coefficient	P value	Coefficient	P value
%ΔT-Bil	−20.0%	−20.0%	−20.0%	0.84	−16.7%	−22.2%	0.015	−0.11	0.043	0.09	0.091
%ΔAST	−20.8%	−26.9%	−12.5%	0.0001	−25.0%	−15.4%	0.0026	0.07	0.19	0.03	0.51
%ΔALP	−6.9%	−8.9%	−4.0%	0.051	−11.3%	−1.3%	<0.0001	0.26	<0.0001	0.11	0.038
%Δγ−GTP	−9.7%	−16.3%	−1.5%	<0.0001	−11.2%	−5.7%	0.012	0.16	0.0086	0.07	0.22
%ΔAlbumin	2.6%	5.0%	0.0%	0.0001	3.1%	0.0%	0.011	−0.17	0.003	−0.01	0.78
%ΔeGFR	−7.1%	−4.3%	−10.3%	0.005	−9.4%	−4.7%	0.0037	−0.13	0.024	0.07	0.1862
%ΔBNP	−56.7%	−58.8%	−54.6%	0.62	−66.4%	−47.8%	<0.0001	−0.03	0.59	0.31	<0.0001
%ΔPIIINP	−1.5%	−9.9%	7.2%	<0.0001	−4.7%	2.4%	0.038	N/A		0.16	0.0047
%ΔP4NP 7S	−15.6%	−16.9%	−13.5%	0.045	−20.7%	−8.2%	<0.0001	0.16	0.0047	N/A	

ALP, alkaline phosphatase; AST, aspartate aminotransferase; BNP, brain natriuretic peptide; eGFR, estimated glomerular filtration rate; γ-GTP, γ-glutamyltransferase; PIIINP, N-terminal propeptide of procollagen type III; P4NP 7S, 7S domain of the collagen type IV N-terminal propeptide; T-Bil, total bilirubin.

### Incidences of primary outcome measure and COX regression analyses

**Figure 2:**
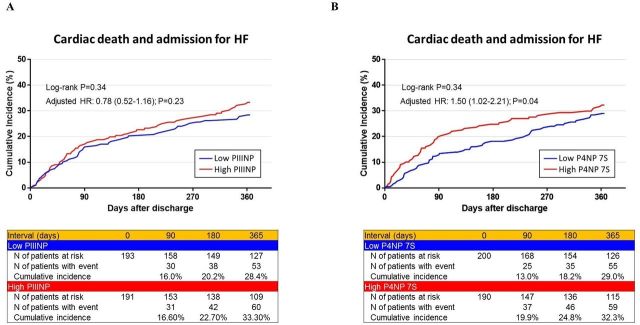
The Kaplan-Meier curves for the primary outcome measure across collagen markers. (A) 365-day primary outcome measure across high PIIINP versus low PIIINP. (B) 365-day primary outcome measure across high P4NP 7S versus low P4NP 7S. (C) 90-day landmark analysis of the primary outcome measure across high PIIINP versus low PIIINP. (D) 90-day landmark analysis of the primary outcome measure across high P4NP 7S versus low P4NP 7S. The primary outcome measure was defined as a composite of cardiac death and admission for HF. PIIINP, N-terminal propeptide of procollagen III; P4NP 7S, 7S domain of the collagen type IV N-terminal propeptide; HF, heart failure.

In this study, the follow-up ratio was 99% after 90 days and 97% after 365 days. The cumulative 365 day incidence of the primary outcome measure was not significantly different between the patients with low and high PIIINP nor between those with low and high P4NP 7S (figure 2A, B). However, after adjusting for confounders, high P4NP 7S correlated with significant excess risk relative to low P4NP 7S for the primary outcome measure (table 3, online supplementary table 2). High PIIINP and the tested LFTs did not correlate with significant excess risk for the primary outcome measure (table 3, online supplementary tables 2 and 3).

By landmark analysis, the cumulative incidence of the primary outcome measure within 90 days after discharge in the patients with high P4NP 7S was significantly higher than that in the patients with low P4NP 7S (figure 2D). Even after adjusting for confounders, excess risk of high P4NP 7S relative to low P4NP 7S for the primary outcome measure within 90 days remained significant. By contrast, high P4NP 7S was no longer correlated with significant excess risk for the primary outcome measure beyond 90 days after discharge (figure 2D and table 3). High PIIINP did not correlate with significant excess risk for the primary outcome measure relative to low PIIINP both within 90 days and beyond 90 days (figure 2C and table 3).

**Table 3:** Crude and adjusted HR associated with collagen markers for early (90-day) and late (365-day) clinical outcomes

	PIIINP (high vs low)				P4NP 7S (high vs low)			
	Unadjusted HR (95% CI)	P value	Adjusted HR (95% CI)	P value	Unadjusted HR (95% CI)	P value	Adjusted HR (95% CI)	P value
Primary outcome measure								
Cardiac death and admission for HF	1.19 (0.83 to 1.73)	0.34	0.78 (0.52 to 1.16)	0.23	1.20 (0.83 to 1.73)	0.34	1.50 (1.02 to 2.21)	0.04
Landmark analysis within 90 days	1.09 (0.65 to 1.81)	0.75	0.79 (0.46 to 1.38)	0.42	1.69 (1.02 to 2.86)	0.04	1.89 (1.11 to 3.26)	0.02
Landmark analysis beyond 90 days	1.25 (0.78 to 2.02)	0.35	0.84 (0.50 to 1.40)	0.5	0.96 (0.59 to 1.54)	0.86	1.26 (0.76 to 2.07)	0.37
Secondary outcome measures								
Admission for HF	1.14 (0.77 to 1.69)	0.5	0.71 (0.47 to 1.09)	0.12	1.33 (0.90 to 1.97)	0.15	1.66 (1.11 to 2.51)	0.01
Cardiac death	0.93 (0.41 to 2.08)	0.86	0.76 (0.31 to 1.79)	0.52	1.02 (0.46 to 2.24)	0.97	1.20 (0.51 to 2.76)	0.68
All-cause death	1.38 (0.84 to 2.29)	0.2	1.15 (0.67 to 1.97)	0.62	0.80 (0.49 to 1.32)	0.39	1.07 (0.63 to 1.79)	0.8

In the multivariable Cox proportional hazards models, age, sex, EF<40% and eGFR were incorporated as risk-adjusting variables.

EF, ejection fraction; eGFR, estimated glomerular filtration rate; HF, heart failure.

### Secondary outcome measures

PIIINP did not correlate with significant excess risk for 365-day HF hospitalisation, all-cause death or cardiac death (table 3 and online supplementary figure 1A, C, E). By contrast, high P4NP 7S correlated with markedly high adjusted excess risk for HF hospitalisation relative to low P4NP 7S (table 3 and online supplementary figure 1B), whereas it did not correlate with significant excess risk of cardiac death nor all-cause death (table 3 and online supplementary figure 1D, F).

### Risk discrimination and reclassification

P4NP 7S at discharge was significantly higher in 61 patients who died from cardiac causes or were hospitalised for HF within 90 days of discharge than those in the remaining patients (online supplementary table 4). By contrast, none of the tested LFTs nor PIIINP at discharge were significantly different between the patients with and without 90-day events (online supplementary table 4). %ΔP4NP 7S tended to be smaller in the patients with 90-day events than those in the remaining patients (–11.5% vs −16.1%, p=0.053; online supplementary table 4).

The c-index of the model with conventional risk factors (reference model) for cardiac death and HF hospitalisation at 90 days postdischarge was 0.654 (table 4). When added to the reference model, none of the individual LFTs at discharge markedly improved the discrimination (table 4). Likewise, regardless of being a categorical or continuous variable, the addition of discharge PIIINP to the reference model did not result in marked improvement in the risk discrimination (table 4). Although the addition of discharge P4NP 7S to the reference model enhanced the c-index by 0.021, the highest increase among tested markers, the improvement was not statistically significant. Of note, the addition of P4NP 7S markedly improved the risk reclassification and discrimination (p=0.038 for NRI; p=0.0068 for IDI).

**Table 4:** The prognostic utility of liver function tests, PIIINP and P4NP 7S

	C-index	ΔC-index	P	NRI (95% CI)	P	IDI (95% CI)	P
Reference model	0.654 (0.579–0.728)						
Reference model +T-Bil	0.668 (0.596–0.741)	0.014	0.43	0.207 (–0.037 to 0.452)	0.096	0.0097 (–0.0041 to 0.024)	0.17
Reference model +AST	0.653 (0.578–0.729)	–0.001	0.97	–0.0325 (–0.251 to 0.186)	0.77	0.0068 (–0.0011 to 0.0021)	0.55
Reference model +ALP	0.653 (0.579–0.727)	–0.001	0.97	0.0659 (–0.168 to 0.30)	0.58	0.0016 (–0.0039 to 0.0071)	0.56
Reference model + γ-GTP	0.652 (0.578–0.727)	–0.002	0.57	–0.104 (–0.362 to 0.155)	0.43	0.000272 (0.00245 to 0.00217)	0.85
Reference model +PIIINP (high/low)	0.657 (0.583–0.731)	0.003	0.76	–0.063 (–0.336 to 0.209)	0.65	0.0022 (–0.0035 to 0.008)	0.45
Reference model +PIIINP (continuous)	0.663 (0.589–0.738)	0.009	0.47	0.112 (–0.157 to 0.382)	0.41	0.007 (–0.0012 to 0.0152)	0.095
Reference model +P4 NP 7S (high/low)	0.675 (0.60–0.751)	0.021	0.68	0.285 (0.016 to 0.555)	0.038	0.019 (0.0052 to 0.033)	0.007
Reference model +P4 NP 7S (continuous)	0.671 (0.599–0.743)	0.017	0.33	0.382 (0.112 to 0.652)	0.006	0.011 (0.00109 to 0.0209)	0.041

The reference model included age, sex, EF<40%, eGFR, sodium <140 mmol/L, haemoglobin and BNP.

ALP, alkaline phosphatase; AST, aspartate aminotransferase; BNP, brain natriuretic peptide; EF, ejection fraction; eGFR, estimated glomerular filtration rate; γ-GTP, γ-glutamyltransferase; IDI, integrated discrimination improvement; NRI, net reclassification improvement; PIIINP, N-terminal propeptide of procollagen type III; P4NP 7S, 7S domain of the collagen type IV N-terminal propeptide; T-Bil, total bilirubin.

## Discussion

The main findings in the present study were (1) P4NP 7S declined in parallel to a decline in BNP, whereas PIIINP did not markedly change during hospitalisation; (2) patients discharged with high P4NP 7S were at high risk of HF readmission, especially within 90 days of discharge; and (3) P4NP 7S, when added on the established risk factors better improved the prediction of early cardiac death and HF hospitalisation than conventional LFTs and PIIINP.

Congestion is the major clinical manifestation in most patients with ADHF.^1^ Increased central venous pressure due to systemic congestion could cause passive organ congestion in the abdominal cavity.^2 11^ In the liver, congestion causes impairment of the physiological circulation, sinusoidal fenestrate enlargement and diminished delivery of oxygen and nutrients, markedly damaging hepatocytes.^11^ Reportedly, hepatic injury with elevated LFTs is among the leading biochemical abnormalities found in patients with ADHF with the reported incidence of 20%–30%.^2 12 13^

Organ congestion could cause fibrosis.^14^ To date, circulating collagen peptides produced during the remodelling of hepatic extracellular matrix (ECM) have been comprehensively investigated as potential markers of liver fibrosis in the primary liver diseases.^15^ Among those, serum levels of PIIINP and P4NP 7S reportedly increase with strong correlations with the histological degree of liver fibrosis in patients with chronic viral liver disease.^16–18^ Previously, we studied the correlation between P4NP 7S and haemodynamic parameters in patients with HF and found that P4NP 7S markedly correlates with pulmonary capillary wedge pressure and right-side cardiac pressure, such as right atrial and ventricular pressure, but not with the cardiac index.^7^ Concordantly, other studies recently reported that P4NP 7S correlated with liver stiffness reflecting right-sided filling pressure in patients with ADHF^19^ and with higher central venous pressure, right-sided volume overload and mortality in patients with pulmonary hypertension.^20^ Although cardiac ECM also expresses collagen types III and IV,^21–24^ our prior research using paired samples of serum and cardiac biopsy tissues reported no marked correlations between the cardiac expression of collagen types III and IV and their corresponding peptides.^6^ In addition, P4NP 7S in the present study correlated with all LFTs and BNP. Taken together, it seems plausible to speculate that elevated P4NP 7S levels in HF reflect accelerated profibrotic response in the liver triggered by congestion-induced injury. However, we could not still deny the possibility that release from extra-cardiac organs other than the liver, such as the lungs^25^ and kidneys,^26^ might contribute to the elevation of circulating collagen peptides; alternatively, serum values of these markers could be affected by the disturbed clearance from the systemic circulation. PIIINP value at discharge was seemingly more affected by renal function. We identified only a weak correlation between discharge PIIINP and P4NP 7S and between %ΔPIIINP and %ΔP4NP 7S, suggesting that the turnover of collagen III and IV could be quite different in their mechanisms.

In this study, we observed that P4NP 7S significantly decreased during hospitalisation with a smaller decline in patients with high P4NP 7S at discharge than in patients with low P4NP 7S. Moreover, high P4NP 7S at discharge was associated with high incidence of HF readmission, with additive prognostic value to conventional prognostic factors. Reportedly, most patients with ADHF experience a marked improvement in clinical congestion during hospitalisation.^27^ However, patients discharged after admission for ADHF enter a vulnerable phase with a very high risk of early readmission.^4^ A subset of patients might have persistent subclinical congestion even at discharge, contributing to early readmission. Although close follow-up, such as an early postdischarge visit, has been recommended for those patients,^28–30^ which subset of patients should be targeted remains uncertain. The evaluation of P4NP 7S might facilitate the identification of those patients at high risk for persistent organ injury and early HF readmission. Of note, none of the abnormal LFTs correlated with independent prognostic significance in this cohort. However, these results were inconsistent with the previous reports showing the prognostic significance of abnormal LFTs in patients with ADHF.^11 12 30^ Notably, all these results were derived from post-hoc analyses of randomised control studies that had mainly targeted patients with advanced HF with decreased EF. In contrast, this study prospectively enrolled consecutive patients with ADHF admitted to the tertiary hospitals. In our population, which well represented patients in the current real-world clinical practice, P4NP 7S was proven superior to conventional LFTs or PIIINP for the prognostic utility.

This study has several limitations. First, peripheral biomarkers of collagen turnover might be affected by conditions other than HF. Although we carefully excluded patients with severe comorbidities, other undetected factors might have affected the values of collagen markers. Second, data regarding the severity of congestion, such as jugular venous pressure, oedema, weight changes during hospitalisation and inferior vena cava diameter, are lacking. Although P4NP 7S exhibited significant correlation with severity of haemodynamic congestion and right-sided pressure in the previous studies,^6^ whether this correlation was also true in the present cohort remains unclear. Third, number of patients in this cohort might still be inadequate for full adjustment by the array of confounders or more detailed analyses in the subgroups of the population. Hence, this study warrants further large-scale study to validate its findings.
