# Supplementary material for: Utility of collagen-derived peptides as markers of organ injury in patients with acute heart failure
Source: Open Heart. 2020 Apr 5;7(1):e001041. doi: 10.1136/openhrt-2019-001041 (PMC7174059; doi:10.1136/openhrt-2019-001041)
Supplement: Supplementary data [file openhrt-2019-001041supp001.pdf]

SUPPLEMENTAL MATERIAL

The utility of collagen-derived peptides as markers of organ injury in patients with acute heart failure: A prospective cohort study

Nagao et al. Collagen markers in acute heart failure

Kazuya Nagao, MD, PhD; Akinori Tamura, MD; Yukihito Sato, MD, PhD; Reo Hata, MD; Yuichi Kawase, MD; Kazushige Kadota, MD, PhD; Takahiro Horie, MD, PhD; Naoya Sowa; Masataka Nishiga, MD, PhD; Koh Ono, MD, PhD; Tsukasa Inada, MD, PhD; Masaru Tanaka, MD, PhD

Supplemental Methods

Participating centers

Osaka Red Cross Hospital, Osaka, Japan,

Hyogo Prefectural Amagasaki General Medical Center, Amagasaki, Japan,

Kurashiki Central Hospital, Kurashiki, Japan

Measurements of markers

We used commercially available radioimmunoassay kits for PIIINP (CIS Bio International, Codolet, France) and P4NP 7S (Sceti Medical Labo, Tokyo, Japan) assays; the lower limits of detection for PIIINP and P4NP 7S were 0.2 U/mL and 1.25 ng/mL, respectively, and the coefficient of variation was <5%. When necessary, the serum was stored at ≤–30°C until assayed following the established protocol used in the clinical setting. Based on the nationwide standardization of laboratory measurements for medical care in Japan (Multianalyte Conventional Reference Material [MacRM]),[17] the following were used as cutoff values of normal LFTs: total bilirubin (T-Bil), 1.5 mg/dL; aspartate aminotransferase (AST), 30 IU/L; alkaline phosphatase (ALP), 322 IU/L; γ-glutamyltransferase (γ-GTP), 64 IU/L in males and 32 IU/L in females. We calculated %changes of (%Δ) markers during hospitalization as (discharge value – admission value)/admission value×100.

Supplemental Table 1. Correlation of PIIINP and P4NP 7S with clinical parameters

Variables	PIIINP		P4NP 7S	
	Coefficient	*P*	Coefficient	*P*
T-Bil, mg/dl	–0.2	0.0001	0.4	<0.0001
AST, U/L	–0.12	0.016	0.28	<0.0001
ALP, U/L	0.073	0.16	0.16	0.002
γ-GTP, U/L	–0.14	0.011	0.41	<0.0001
Albumin, g/dl	–0.1	0.059	0.045	0.38
eGFR, mL/min/1.73 m^2	–0.44	<0.0001	–0.07	0.0063
BNP, pg/ml	0.095	0.071	0.25	<0.0001
PIIINP, U/mL	N/A		0.24	<0.0001
P4NP 7S, ng/mL	0.24	<0.0001	N/A	

ALP, Alkaline phosphatase; AST, aspartate aminotransferase; BNP, brain natriuretic peptide; eGFR, estimated glomerular filtration rate; γ-GTP, γ-glutamyltransferase; PIIINP, N-terminal propeptide of procollagen type III; P4NP 7S, 7S domain of the collagen type IV N-terminal propeptide; T-Bil, total bilirubin.

Supplemental Table 2. Univariate and multivariate COX analyses of the 365-day incidence of cardiac death and admission for HF

Variable	Unadjusted HR (95%CI)	*P*	Adjusted HR (95%CI)	*P*	Adjusted HR (95%CI)	*P*
			Model 1		Model 2	
Age (per 1 year)	1.02 (1.00-1.04)	0.04	1.01 (0.99-1.03)	0.44	1.01 (0.99-1.03)	0.2
Male	1.14 (0.79-1.66)	0.48	1.39 (0.94-2.05)	0.1	1.37 (0.93-2.02)	0.11
EF <40%	0.84 (-.57-1.22)	0.36	1.02 (0.68-1.52)	0.9	1.04 (0.69-1.55)	0.86
eGFR (per 1 mL/min/1.73 m^2)	0.97 (0.96-0.98)	<0.001	0.97 (0.96-0.98)	<0.001	0.97 (0.96-0.98)	<0.001
PIIINP (high versus low)	1.19 (0.83-1.73)	0.34	**0.78 (0.52-1.17)**	**0.23**	-	
P4NP 7S (high versus low)	1.20 (0.83-1.73)	0.34	-		**1.50 (1.02-2.21)**	**0.04**

EF, ejection fraction; HF, heart failure; HR, hazard ratio

Supplemental Table 3. Crude and adjusted HR associated with abnormal LFTs for cardiac death and admission for HF

	Unadjusted HR (95% CI)	*P*	Adjusted HR (95% CI)	*P*
T-Bil	1.26 (0.44–2.78)	0.63	1.58 (0.55–3.57)	0.36
AST	0.72 (0.42–1.15)	0.17	0.86 (0.50–1.40)	0.56
ALP	1.17 (0.73–1.79)	0.51	1.37 (0.86–2.12)	0.18
γ-GTP	0.70 (0.45–1.05)	0.08	0.89 (0.56–1.39)	0.62

In the multivariable Cox proportional hazards models, age, male, ejection fraction (EF) <40%, and estimated glomerular filtration rate (eGFR), were incorporated as risk-adjusting variables. ALP, alkaline phosphatase; AST, aspartate aminotransferase; γ-GTP, γ-glutamyltransferase; T-Bil, total bilirubin.

Supplemental Table 4. Difference in markers at discharge and %Δmarkers during hospitalization between the patients with and without 90-day events

	Markers at discharge			%Δmarkers		
	90-day cardiac death and HF hospitalization		*P*	90-day cardiac death and HF hospitalization		*P*
	No	Yes		No	Yes	
T-Bil	0.6 (0.5–0.9)	0.7 (0.5–0.9)	0.43	-20.0% (-37.5–0)	-22.2% (-38.7–1.8)	0.84
AST	22 (17–29)	23 (17–28)	0.68	-29.0% (-88.6–0)	-12.0% (-42.2–13.2)	0.0037
ALP	237 (195–303)	225 (171–309)	0.31	-7.1% (-18.9–5.3)	-1.8% (-17.1–11.5)	0.31
γ-GTP	33 (19–67)	26 (17–87)	0.50	-10.4% (-29.0–9.1)	-3.8% (-18.9–11.6)	0.09
PIIINP	0.71 (0.57–0.93)	0.72 (0.61–0.90)	0.52	-0.5% (-12.9–16.4)	-5.1% (-17.9–10.0)	0.13
P4NP 7S	5.0 (4–6.4)	5.6 (4.4–7.1)	0.042	-16.1% (-27.1–-5.4)	-11.5% (-26.1–3.5)	0.053

ALP, alkaline phosphatase; AST, aspartate aminotransferase; γ-GTP, γ-glutamyltransferase; HF, heart failure; PIIINP, N-terminal propeptide of procollagen type III; P4NP 7S, 7S domain of the collagen type IV N-terminal propeptide; T-Bil, total bilirubin.

Supplemental Figure 1

A

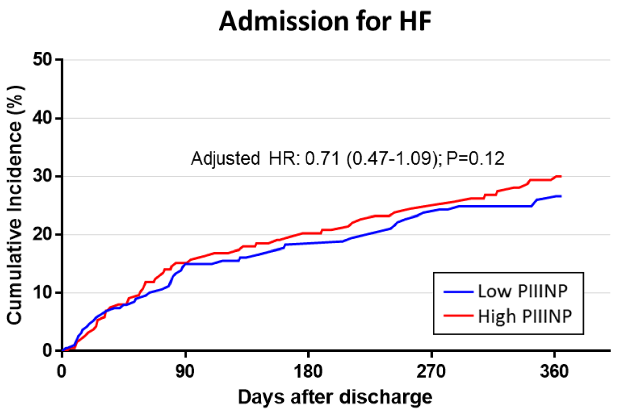

Interval	0 day	90 days	180 days	365 days
Low PIIINP				
N of patients at risk	193	158	148	127
N of patients with event		28	34	49
Cumulative incidence		15.0%	18.3%	26.6%
High PIIINP				
N of patients at risk	191	152	137	109
N of patients with event		28	37	53
Cumulative incidence		15.2%	20.3%	30.0%

B

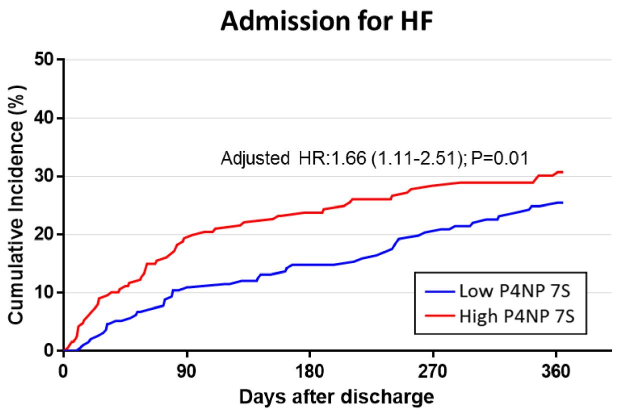

Interval	0 day	90 days	180 days	365 days
Low P4NP 7S				
N of patients at risk	200	167	154	126
N of patients with event		21	28	47
Cumulative incidence		11.0%	14.8%	25.5%
High P4NP 7S				
N of patients at risk	190	147	136	115
N of patients with event		36	44	56
Cumulative incidence		19.4%	23.8%	30.8%

C

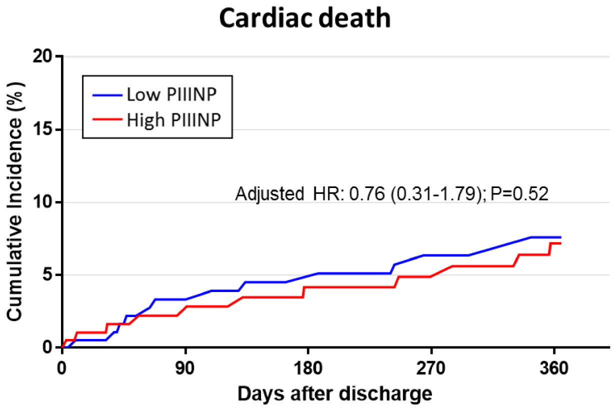

Interval	0 day	90 days	180 days	365 days
Low PIIINP				
N of patients at risk	193	165	158	143
N of patients with event		6	8	13
Cumulative incidence		3.3%	4.5%	7.6%
High PIIINP				
N of patients at risk	191	152	137	109
N of patients with event		4	7	11
Cumulative incidence		2.8%	4.2%	7.2%

D

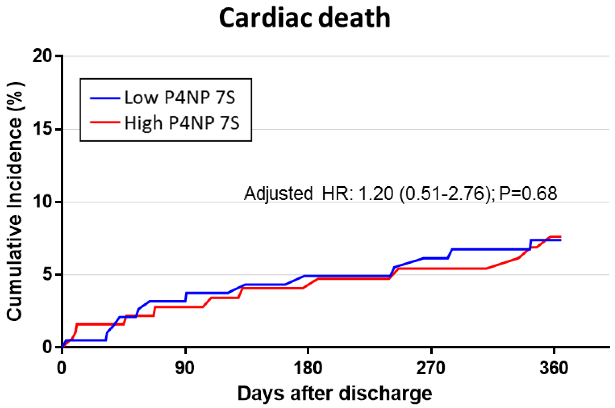

Interval	0 day	90 days	180 days	365 days
Low P4NP 7S				
N of patients at risk	200	167	154	126
N of patients with event		6	9	13
Cumulative incidence		3.2%	4.9%	7.4%
High P4NP 7S				
N of patients at risk	190	147	136	115
N of patients with event		5	7	12
Cumulative incidence		2.8%	4.1%	7.6%

1 **E**

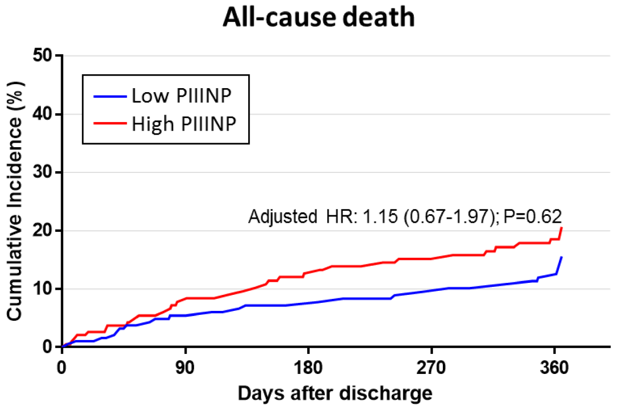

Interval	0 day	90 days	180 days	365 days
Low PIIINP				
N of patients at risk	193	165	158	143
N of patients with event		11	14	28
Cumulative incidence		6.0%	7.7%	16.1%
High PIIINP				
N of patients at risk	191	155	142	115
N of patients with event		15	23	35
Cumulative incidence		8.3%	13.1%	21.1%

F

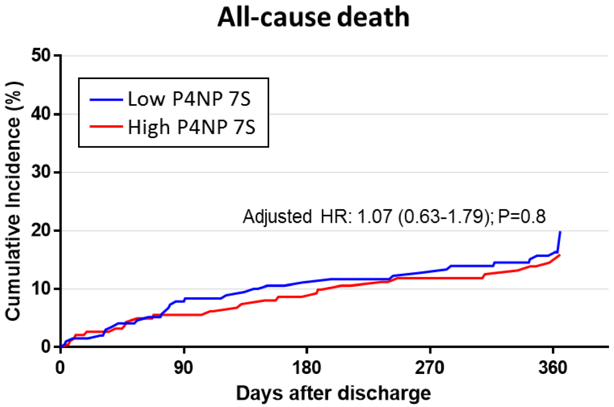

Interval	0 day	90 days	180 days	365 days
Low P4NP 7S				
N of patients at risk	200	170	160	138
N of patients with event		16	22	37
Cumulative incidence		8.3%	11.6%	20.4%
High P4NP 7S				
N of patients at risk	190	155	145	125
N of patients with event		11	16	27
Cumulative incidence		6.1%	9.1%	16.4%

Supplemental Figure Legends

Supplemental Figure 1. The Kaplan–Meier curves for secondary outcome measures across PIIINP (A, C and E) and P4NP 7S (B, D and F).

The seconday outcome measures were defined as admission for HF (A and B), cardiac death (C and D) and all-cause death (E and F). HF, heart failure; HR, hazard ratio; PIIINP, N-terminal propeptide of procollagen III; P4NP 7S, 7S domain of the collagen type IV N-terminal propeptide.
